# Low-Cycle Fatigue Behavior of 10CrNi3MoV High Strength Steel and Its Undermatched Welds

**DOI:** 10.3390/ma11050661

**Published:** 2018-04-24

**Authors:** Wei Song, Xuesong Liu, Filippo Berto, Seyed Mohamad Javad Razavi

**Affiliations:** 1 State Key Laboratory of Advanced Welding and Joining, Harbin Institute of Technology, Harbin 150001, China; swingways@hotmail.com; 2 Department of Mechanical and Industrial Engineering, Norwegian University of Science and Technology (NTNU), 7491 Trondheim, Norway; javad.razavi@ntnu.no

**Keywords:** 10CrNi3MoV steel, undermatched welds, low cycle fatigue, cyclic deformation behavior, strain energy density

## Abstract

The use of high strength steel allows the design of lighter, more slender and simpler structures due to high strength and favorable ductility. Nevertheless, the increase of yield strength does not guarantee the corresponding improvement of fatigue resistance, which becomes a major concern for engineering structure design, especially for the welded joints. The paper presents a comparison of the low cycle fatigue behaviors between 10CrNi3MoV high strength steel and its undermatched weldments. Uniaxial tension tests, Push-pull, strain-controlled fatigue tests were conducted on base metal and weldments in the strain range of 0.2–1.2%. The monotonic and cyclic stress-strain curves, stress-life, strain-life and energy-life in terms of these materials were analyzed for fatigue assessment of materials discrepancy. The stress-life results of base metal and undermatched weld metal exhibit cyclic softening behaviors. Furthermore, the shapes of 10CrNi3MoV steel hysteresis loops show a satisfactory Masing-type behavior, while the weld metal shows a non-Masing type behavior. Strain, plastic and total strain energy density amplitudes against the number of reversals to failure results demonstrate that the undermatched weld metal presents a higher resistance to fatigue crack initiation than 10CrNi3MoV high strength steel. Finally, fatigue fracture surfaces of specimens were compared by scanning electron microscopy to identify the differences of crack initiation and the propagation between them.

## 1. Introduction

Modern steel manufacturing techniques make it easier to produce high strength steel for various fields of engineering structure applications, such as shipbuilding, marine structures, engineering machinery, bridges, and so on [1,2]. Although it has a bright future for engineering applications due to high yield strength, favorable toughness, good weldability and cost efficiency, these properties improvement do not guarantee the corresponding enhancement of fatigue resistance. Especially for the welded joints, the toughness will be decreased unavoidably after welding when the strength of welded joints keep the same level with base metal. Usually, it needs to sacrifice weld metal strength to enhance the ductility of welded joints. 10CrNi3MoV high strength steel is one of the reliable materials for key components in shipbuilding due to its superior mechanical properties and good weld ability [3]. So far, the welding process of this steel has been widely investigated by some researchers. The influence of different welding conditions on varied heat affected zone (HAZ) by double-sided double gas tungsten arc welding (DSGTAW) was studied by microstructure observations by Peng et al. [4,5,6]. Its corrosion properties related to fatigue behavior and hydrostatic pressure have also been examined explicitly [7,8]. However, the fatigue behavior of 10CrNi3MoV steel and its corresponding welds is still a major concern for structure design due to the variation of material properties.

Since it reported that the fatigue resistance of high strength steel does not boost proportionally with the static strength improvement [9]. Meanwhile, fatigue properties are one of the most important considerations for service reliability assessment of welded structures, Although there are some local methods to be used to assess the fatigue strength of welded joints on steel and other alloys [10,11,12,13,14], it is necessary to access and compare the difference of fatigue resistance between base metal and corresponding weld metal. For the welded joints made by high strength steel, the fatigue behavior may be lower than that made by mild strength steel [15]. There are a great number of references to investigate the fatigue properties of homogeneous materials under low cycle fatigue. The effects of stain amplitude [16], strain rate [17], tungsten addition [18], temperature [19] on microstructure and fatigue behaviors have been examined systematically according to material application backgrounds by researchers in recent years. Branco et al. [20,21] performed low-cycle fatigue (LCF) tests on the 34CrNiMo6 high strength steel and DIN 34CrNiMo6 tempered alloy steel to study the comprehensive fatigue properties, which contain cyclic deformation response, fatigue strength, fatigue ductility properties and fatigue mechanism. The fatigue crack initiation and growth properties of high strength steel S690 and mild strength steel S355 were compared by smooth specimens low cycle fatigue tests and CT specimens fatigue crack growth experiments [22]. The results found that the high strength steel had superior resistance of fatigue crack initiation, while the crack propagation rates increased with the increases of yield strength. Recently, Veerababu et al. [19] reported LCF properties of Grade 92 steel base metal and its even-matched welded joints considering various high temperatures. The results showed that the welds have lower cyclic stress response than its base metal. Similarly, the studies from Westerbaan et al. [23] and Xu et al. [24] showed that High Strength Low Alloy (HSLA) steel laser welds had lower fatigue resistance than base metal under load-controlled at high stress amplitudes. Sowards et al. [25] conducted low cycle fatigue experiments for butt welds of HSLA. It revealed that weld fatigue susceptibility was higher than base metal at higher strain amplitudes, although weld cyclic strength was greater than that of base metal resulting in lower plastic strains. Nevertheless, limited literatures pay attention to LCF properties of undermatched welded joints. 

In general, low cycle fatigue life for materials and welded joints under uniaxial and multiaxial cycle loading is assessed by the strain-life methods [26], or energy methods [27,28,29,30]. The energy parameters can effectively reflect the comprehensive relationship between the stress and strain under cyclic loading. Therefore, the paper aims to study the effect of yield strengths of base metal and weld metal on the LCF properties based on the energy method. It mainly provides experimental assessment of the LCF behaviors of 10CrNi3MoV high strength steel and corresponding undermatched weldments. The paper is organized as follows. Section 2, LCF tests are conducted under full-reversed strain-controlled conditions with the amplitudes between 0.2% and 1.2% at room temperature. Subsequently, the cyclic deformation behaviors, the cyclic stress-strain response of welded joints, the failure locations in the welded joints are analyzed and compared with base metal in Section 3. In the same section, the different elastic and plastic deformation relationships are determined according to Coffin, Manson and Basquin equations from the experimental data. Finally, the fatigue fractographic of these materials were examined to understand the failure phenomena by scanning electron microscopy (SEM).

## 2. Experimental Procedure

**Figure 1:**
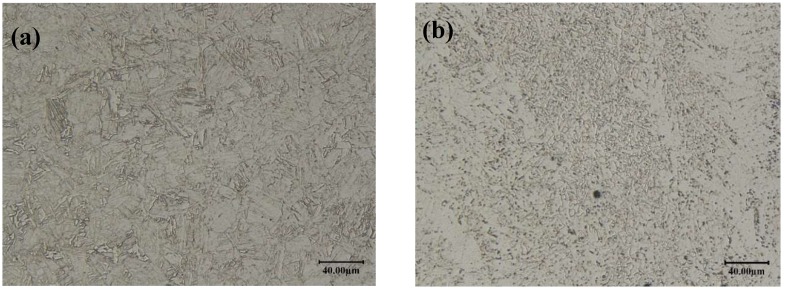
Microstructures of the investigated materials: (**a**) 10CrNi3MoV high strength steel; (**b**) undermatched welds.

The 10CrNi3MoV high strength steel plate of 16 mm thick rolled was received in the quenched and tempered condition. Its corresponding undermatched welds were processed by gas metal arc welding (GMAW) with multiple passes using undermatched electrodes. Table 1 outlines the nominal chemical composition of these materials from the manufacturers’ certificate. The microstructures of 10CrNi3MoV high strength steel and its undermatched weldments were observed using an optical microscope. Figure 1 compares the microscopes difference of these materials. The micrographs illustrate the grain boundaries and the non-uniform distribution of precipitates. 10CrNi3MoV high strength low alloy steel, which is quenched and tempered, has a fine microstructure mainly composed of acicular ferrite and granular carbides. For the weld metal after high speed cooling, it is observed that smaller ferrite needles are embed into the martensite grains.

**Figure 2:**
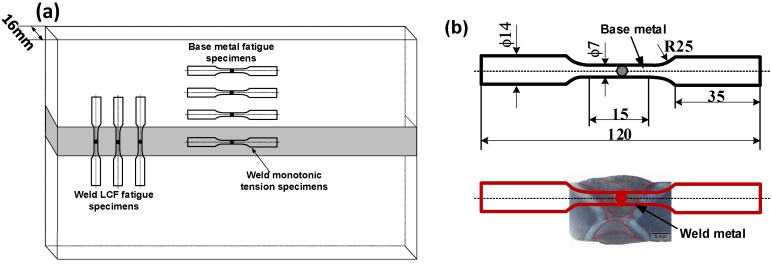
(**a**) Orientation of monotonic tension and fatigue test specimens with respect to the welded joints; (**b**) Schematic of LCF test specimens about base metal and weld metal.

All the smooth fatigue test specimens were machined and processed from a 500 mm × 300 mm × 16 mm welding plate. Table 2 provides the plate multi-pass welding conditions using the GMAW process. The extracted locations of base metal and weld metal smooth specimens are depicted in Figure 2a. It should be noted that the extracted direction of weld metal specimens is vertical to welding bead direction. The width of weld bead is in the range from 5 mm to 10 mm, which does not exceed the limited range of extensometer. The location relationship between the butt weld joint and extracted specimens is shown in Figure 2b. It means that the fatigue failure may occur in HAZ or fusion zone. The judgement of failure location will be presented in the next section. The geometry of smooth LCF cylindrical specimens is also shown in Figure 2b. The gauge length of specimens was polished by an appropriate sequence of sandpapers. The LCF tests for base metal and welds were conducted as ASTM E606 standard [31] in INSTRON 8802 electromechanical fatigue testing machine in the air environment. The strain amplitude was controlled using a dynamic clip gauge of INSTRON. The specimens of these materials were instrumented with a reference gauge length of 12.5 mm for the fatigue tests. In order to investigate the effect of strain amplitude on fatigue mechanical response, these LCF tests were carried out at strain amplitudes varying from ±0.2 to ±1.2% under full reverse sinusoidal waveform with total axial strain control mode.

Optical and scanning electron microscopic investigation was conducted to understand the microstructural and fracture appearance difference after fatigue tests between the base metal and weldments.

## 3. Results

### 3.1. Monotonic Tensile Results and Micro-Hardness Analysis

**Figure 3:**
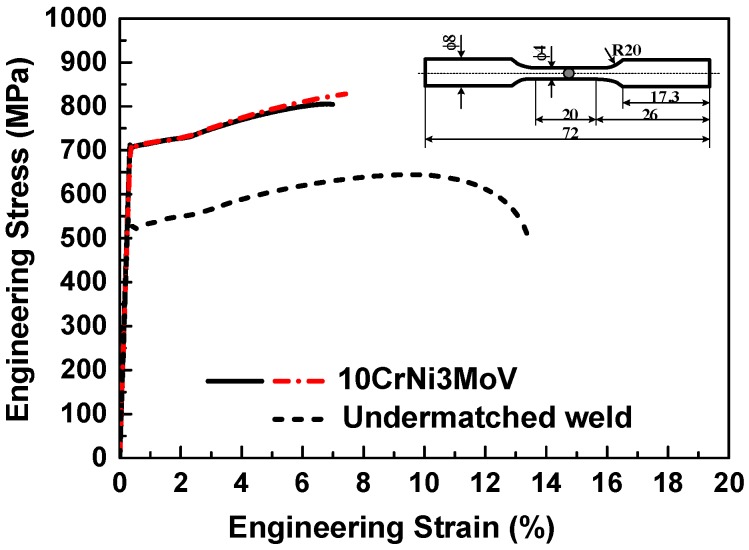
Monotonic stress-strain curves of 10CrNi3Mov high strength steel and its undermatched welds.

Figure 3 shows the monotonic stress-strain curve under the strain rate of $2\times 10^{-3}/s$. As seen from the results, the ratio between the 0.2% proof yield strength (*σ_YS_*) and the ultimate tensile strength (*σ_UTS_*) is high for 10CrNi3MoV steel, which is 1.07. It means that the capability for hardening is limited. Compared with base metal, the undermatched welds have a higher T/Y ratio, which is 1.12. The mechanical properties of these materials under monotonic tension loading are summarized in Table 3.

**Figure 4:**
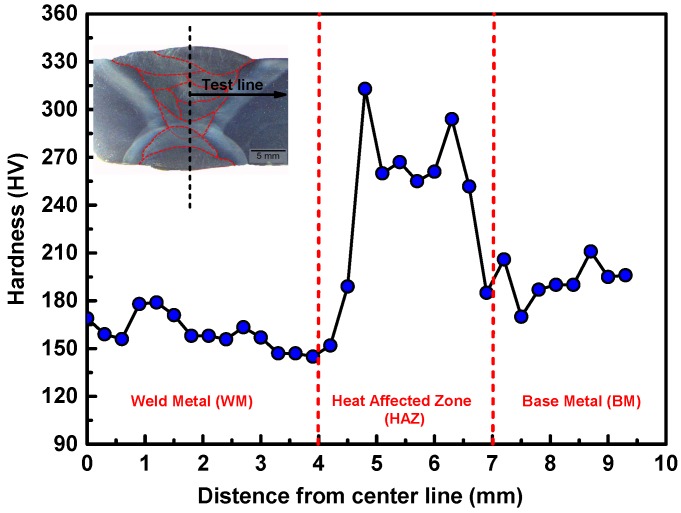
Microhardness profile of the welded joint.

Figure 4 depicts the Vickers microhardness profile of the undermatched welded joint. The average hardness of BM and WM was about 200 and 160 HV, respectively. The hardness of HAZ was distributed from 315 to 270 HV. According to the hardness distribution of welded joints, it shows that fusion region (weld metal) is the weakest zone of the whole joint.

### 3.2. Fatigue Tests Results

Table 4 summarized the results of fatigue test carried out with smooth specimens for base metal and its undermatched welds under strain-controlled conditions. The table includes the controlled total strain amplitudes, elastic and plastic strain amplitudes, responding stress amplitudes, plastic strain energy density, total strain energy density and the resulting number of cycles to failure, *N_f_*, for each specimen. The stabilized hysteresis loops were used to determine the plastic strain and corresponding energy density values directly.

Given the size of the fatigue specimen (Φ7 mm), a great proportion of welding residual stress has been released in the cutting process of specimens. On the other hand, a considerable residual stress relaxation occurs during the beginning fatigue cycles under large loading in low cycle fatigue. Thus, the residual stress can be neglected in low cycle fatigue.

**Figure 5:**
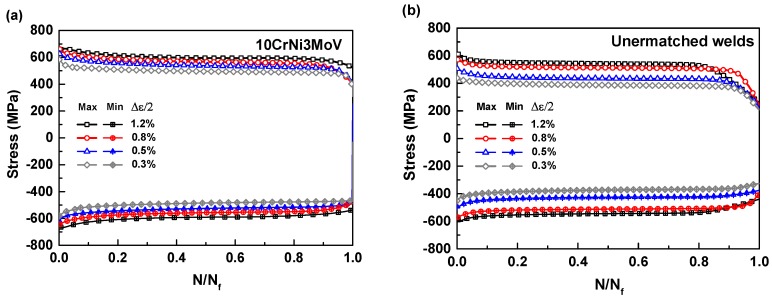
Evolution of the maximum and minimum stress with life ratio for different values of strain amplitudes: (**a**) 10CrNi3MoV steel; (**b**) Undermatched welds.

The influence of total strain amplitudes on cyclic stress response (Maximum stress and Minimum stress) against normalized life ratio of 10CrNi3MoV steel and its undermatched welds are depicted in Figure 5a,b, respectively. Regardless of the strain amplitude, the obvious cyclic strain-softening behaviors can be observed from the base metal and undermatched weld metal plots. As can be seen from these figures, the cyclic stress variation composes a rapid initial softening at the beginning 5–15% cyclic stage. Then keeps stable stress responses until 90–95% of life ratio and experiences a rapid drop in stress culminating in fatigue failure finally. Therefore, the stable stress and strain magnitudes at half-life can be used to assess the fatigue behavior of materials. These results further illustrate the effectiveness of parameters from stabilized half-life cyclic stress-strain hysteresis loops. Since the lower cycle strength of undermatched welds than base metal, the cycle stress responses also exhibit the corresponding decreasing under the same strain amplitudes. The softening behaviors of these materials verified the empirical rule proposed in [32], in which metals with $\sigma_{UTS}/\sigma_{YS}<1.2$ demonstrated cyclically soften (10CrNi3MoV steel $\sigma_{UTS}/\sigma_{YS}=1.07$ and undermatched welds $\sigma_{UTS}/\sigma_{YS}=1.12$). Additionally, the degree of softening is decreased with the increases of strain amplitudes.

**Figure 6:**
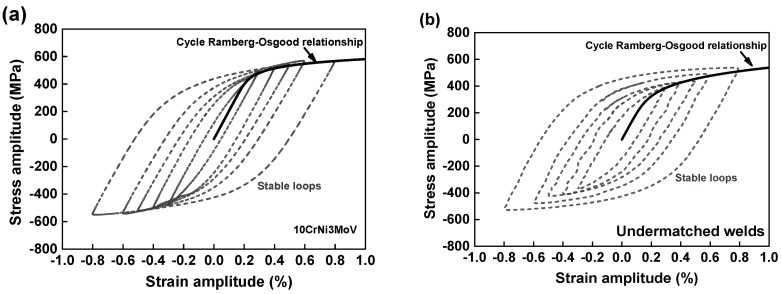
Stabilized stress-strain hysteresis loops and cycle Ramberg-Osgood relationships under different amplitudes: (**a**) 10CrNi3MoV steel; (**b**) Undermatched welds.

Figure 6 presents the stabilized cyclic stress-strain hysteresis loops using a half-life criterion obtained from these materials fatigue results. It is clear that 10CrNi3MoV steel shows a great consistency with increases of strain amplitudes from Figure 6a. While the cyclic stress-strain hysteresis of weld metal in Figure 6b demonstrates some difference with the base metal, the peak cyclic stress response cannot be superimposed under different strain levels. Based on the combination of hysteresis curves under different strain amplitudes, the stabilized cycle Ramberg-Osgood relationships are determined by the fitting of each maximum cyclic stress. Generally, the Masing behaviors can be observed if the upper branches of the hysteresis loops are all coincident. For a material obeying the Masing-type behavior, the relationship between cyclic stress and plastic strain amplitudes, the plastic strain energy density variation may be illustrated by the cyclic curves of the material. Thus, the shape of the hysteresis loops of 10CrNi3MoV steel exhibits a satisfactory Masing-type behavior. Whereas the undermatched welds do not have the characteristics of Masing behavior, being a non-Masing material.

**Figure 7:**
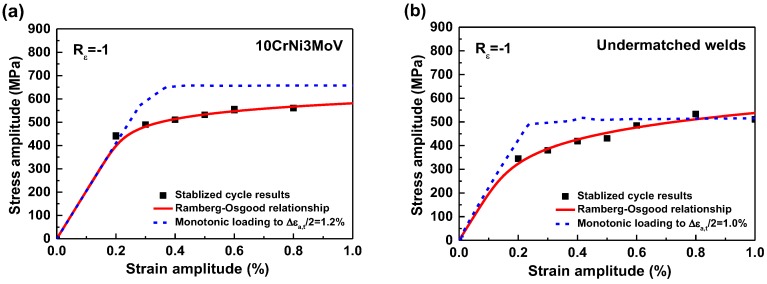
Stress-strain comparison between monotonic tension and cycle Ramberg-Osgood relationships: (**a**) 10CrNi3MoV steel; (**b**) Undermatched welds.

Figure 7a depicts the stress-strain comparison between monotonic tension and cyclic Ramberg-Osgood relationships for 10CrNi3MoV steel. The results show that stress in the plastic stage from cyclic curves is lower than the monotonic stress-strain curves due to the material cyclic softening behavior. Similarly, the weld metal cyclic stress-strain curves also indicate the stress declining tendency in Figure 7b. After that, the parameters of different cyclic stress-strain curves are computed according to Equation (1), which are summarized in Table 5.

(1) $$\Delta \varepsilon =\frac{\Delta \sigma }{E}+2{\left(\frac{\Delta \sigma }{2K^{\prime }}\right)}^{1/n^{\prime }}$$

### 3.3. The Analysis of Hysteresis Loops

Although the cyclic stress-strain curve describes the relationship between stable stress and strain amplitudes, it cannot acquire some analytical information about the shape of hysteresis loop branches. As mentioned above, 10CrNi3MoV steel exhibits a good Masing behavior, while weld metal shows a non-Masing behavior. Hence, the analysis of hysteresis loops may provide a way to understand the microstructural behavior of the material. Generally, the hysteresis loops can be extracted at half-life for materials fatigue results.

The plastic strain energy density per cycle, Δ*W_p_*, is the area of the hysteresis loop. The total damage under fatigue loading, Δ*W_T_*, are the plastic strain energy and the tension part of the elastic strain energy. As for the calculation of Δ*W_p_*, they can be conducted by a “master curve” for both non-Masing and ideal Masing material description [33]. The curve is different from the defined cyclic stress-strain curve. We can match the upper branches of half-life hysteresis loops under many strain amplitudes by translating the locations along its linear response portion. The relationship for the master curve with the origin at the tip of the smallest plastic strain hysteresis loop is proposed as follows:

(2) $$\Delta \varepsilon^{*}=\frac{\Delta \sigma^{*}}{E}+2{\left(\frac{\Delta \sigma^{*}}{2K^{*}}\right)}^{1/n^{*}}$$

**Figure 8:**
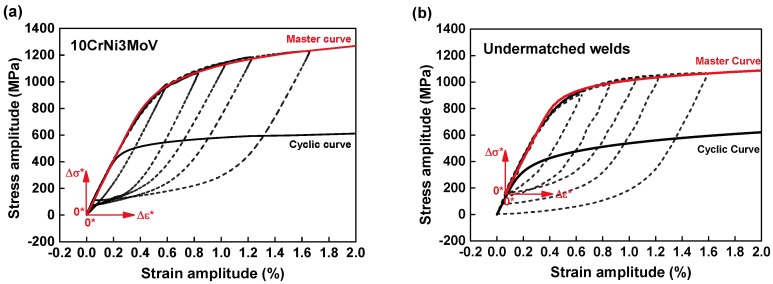
Superposition of the stable hysteresis loops at half-life along the linear portion to match upper branches, and corresponding master curves: (**a**) 10CrNi3MoV steel; (**b**) Undermatched welds.

The hysteresis loops for 10CrNi3MoV steel, Figure 8a, show the good superposition of peak stress values under different strain amplitudes without translating the original coordination. Moreover, the corresponding master curve and the cyclic curve are superimposed for comparison.

Regardless of the hysteresis loops of weld metal, the upper branches of loops do not form a unique continuous curve as a non-Masing behavior. However, translating the original position of hysteresis loops under different strain amplitudes in coordination makes the upper branches superimposed alone a linear response, which is shown in Figure 8b. The original coordination 0 is moved to another position $0^{*}$ by numeric conversion for superimposing. A new auxiliary coordinate system $\left(\Delta \varepsilon^{*},\Delta \sigma^{*}\right)$ with a new origin $0^{*}$ was established. Therefore, the master curve of non-Masing material is obtained from these transitions.

As stated above, the plastic strain energy density (Δ*W_p_*) due to the plastic deformation can be calculated by the area of hysteresis loop. For a Masing-type material, it can be expressed as [34]:

(3) $$\Delta W_{p}=\frac{1-n^{\prime }}{1+n^{\prime }}\Delta \sigma \Delta \varepsilon_{p}$$

where the Δσ is the stress range, $\Delta \varepsilon_{p}$ is the plastic strain range and $n^{\prime }$ is the cyclic hardening exponent. For a non-Masing material, the Δ*W_p_* can be calculated from the equation as following [28]:

(4) $$\Delta W_{p}=\frac{1-n^{*}}{1+n^{*}}\Delta \sigma \Delta \varepsilon_{p}+\frac{2n^{*}}{1+n^{*}}\delta \Delta \sigma_{0}\Delta \varepsilon_{p}$$

where the $n^{*}$ is the hardening exponent of the master curve and $\delta \Delta \sigma_{0}=\Delta \sigma -\Delta \sigma^{*}$.

Summaries of all parameters of cyclic stress-strain curves and master curves for 10CrNi3MoV steel and weld metal are given in Table 6. More discussion of on the results of hysteresis loops in provided in next section.

### 3.4. Low Cycle Fatigue Life

The fatigue assessment of material and components generally classes into S-N, local and fracture mechanics-based approaches. As the global approach, S-N curves method for components and structures are a fundamental method and included in some standard recommendations, such as IIW, Eurocode 3. However, this approach does not account for the material life behaviors. The local approaches can illustrate the local fatigue behavior since it can recognize the localized nature of the fatigue damage. The most well-known relationships are the proposals by Basquin [35], Equation (5) Manson [36] and Coffin [37], Equation (6), and the Morrow [34], Equation (7):

(5) $$\frac{\Delta \sigma }{2}=\sigma_{f}^{\prime }{\left(2N_{f}\right)}^{b}$$

(6) $$\frac{\Delta \varepsilon_{p}}{2}=\varepsilon_{f}^{\prime }{\left(2N_{f}\right)}^{c}$$

(7) $$\frac{\Delta \varepsilon_{t}}{2}=\frac{\Delta \varepsilon_{e}}{2}+\frac{\Delta \varepsilon_{p}}{2}=\frac{\sigma_{f}^{\prime }}{E}{\left(2N_{f}\right)}^{b}+\varepsilon_{f}^{\prime }{\left(2N_{f}\right)}^{c}$$

where the $\sigma_{f}^{\prime }$ and b are the fatigue strength coefficient and exponent, respectively. $\varepsilon_{f}^{\prime }$ and c are the fatigue ductility coefficient and exponent, respectively. The $2N_{f}$ is the number of reversals to failure. $\Delta \varepsilon_{t}$, $\Delta \varepsilon_{e}$ and $\Delta \varepsilon_{p}$ are the total, elastic and plastic strain range, respectively. Δσ is the stress range. *E* is the Young’s modulus.

**Figure 9:**
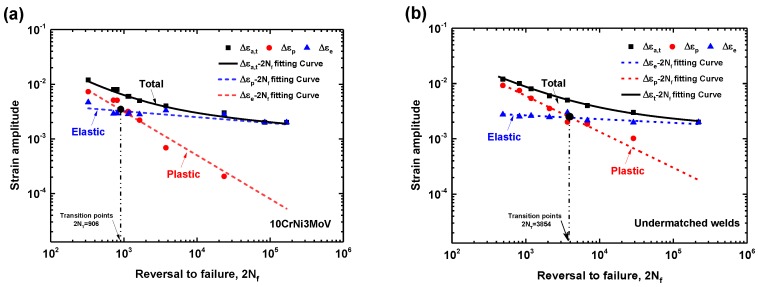
Manson-Coffin curves of test materials: (**a**) 10CrNi3MoV steel; (**b**) Undermatched welds.

**Figure 10:**
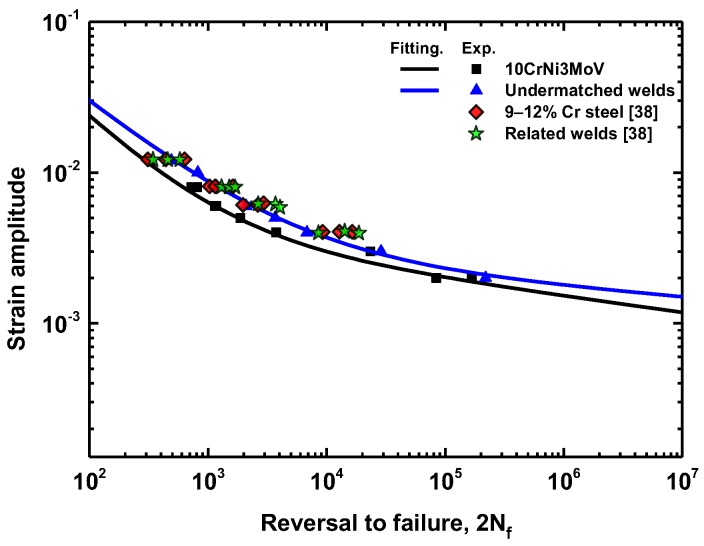
Comparison between Manson-Coffin curves of materials from test and reference [38].

Figure 9 shows the low cycle fatigue life according to Manson-Coffin curves for 10CrNi3MoV steel and its undermatched welds. The analysis of the results shows that the number of transition reversals ($2N_{f}$) is very distinct between the two materials. The base metal has a small number of transition reversals about 1000 cycles in Figure 9a. It means that the fatigue life above 1000 cycles the fatigue behavior of the steel is governed by fatigue strength properties rather than the ductility properties. However, the undermatched welds have a larger number of transition reversals than base metal, which is about 3854 cycles, as shown in Figure 9b. Therefore, the plastic deformation leads to more fatigue damage for the base metal than for its undermatched weld metal. To compare the fatigue properties of these materials directly, the total strain amplitudes against life curves were fitted according to Equation (7). Figure 10 depicts a comparison of total strain amplitudes-life between base metal and undermatched welds. For the final results of strain-life curves, it can be seen that the low strength weld metal demonstrates a stronger fatigue behavior than the high strength base metal for all the range of total strain amplitudes. We also compare LCF results of similar material (10% Cr martensitic steel) and its Ni-based welds from [38] in Figure 10. According to the fatigue ductility and strength-life points of these materials, the BM and WM demonstrate the similar fatigue resistance behavior. However, the base metal (10CrNi3MoV steel) in our study has lower fatigue resistance than the 10% Cr martensitic steel and its Ni-based welds. The undermatched welds show the similar fatigue failure behaviors with 10% Cr martensitic steel. Finally, fatigue strength and fatigue ductility parameters of 10CrNi3MoV high strength steel and undermatched welds are summarized in Table 5.

### 3.5. Energy-Life Relationships

**Figure 11:**
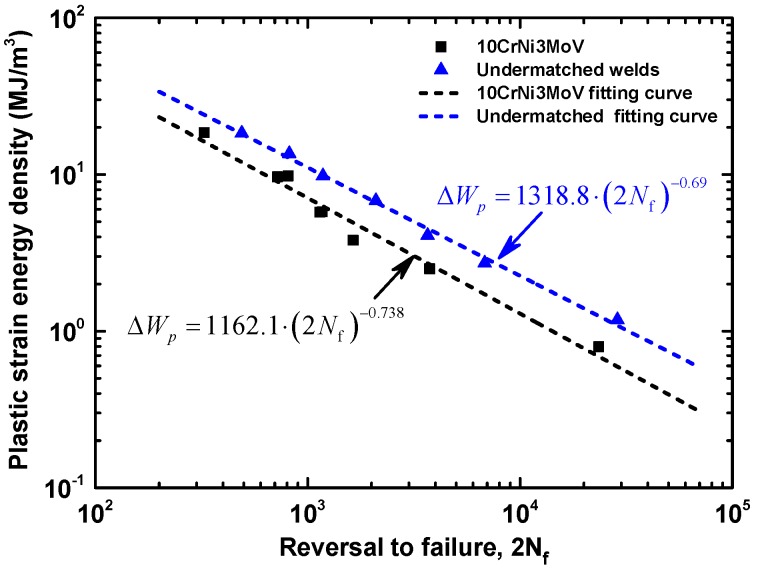
Comparison of Plastic strain energy density Δ*W_p_* between 10CrNi3MoV high strength steel and undermatched welds.

**Figure 12:**
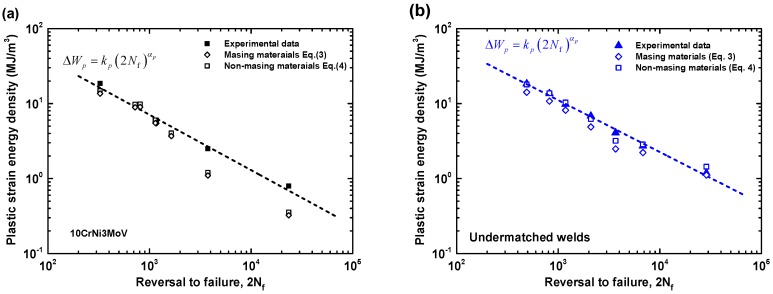
Comparison of Plastic strain energy density Δ*W_p_* from different equations: (**a**) 10CrNi3MoV high strength steel; (**b**) undermatched welds.

**Figure 13:**
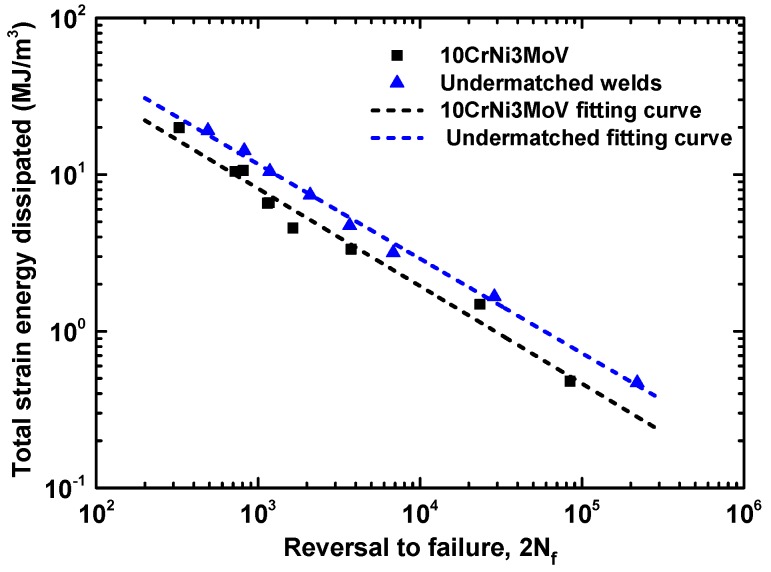
Comparison of total strain energy density Δ*W_t_* between 10CrNi3MoV high strength steel and undermatched welds.

From experimental data of base metal and weld metal cyclic curves in Figure 7, the master curve hardening exponents fitted by the least square method are obtained. Thus, the evolution of $\Delta W_{P}$ at the half-life reversals to failure life (2*N_f_*) can be compared with a log-log scale, which is determined by the measuring area of hysteresis loops, Masing-type Equation (3), and non-masing type Equation (4). Table 6 exhibits the values of $\Delta W_{P}$ with the corresponding strain amplitudes based on different equations. Seen from values in Table 6, the results of different methods are quite close. It is worthy to note that these values can be fitted by a linear relationship. Further, the stable linear relationship realizes the quantity of fatigue life by a proper damage parameter. The evolution of $\Delta W_{P}$ from experiments against fatigue life, which is shown as dashed line in Figure 11, can be fitted by a power law function from Equation (8). Figure 12 shows the comparison of plastic strain energy density Δ*W_p_* between 10CrNi3MoV high strength steel and its undermatched welds. The fitting linear relationship agrees well the experimental data for base metal and weld metal. In this manner, the stable trends give to the quantity the attribute of a proper fatigue damage parameter for fatigue assessment. From the results in Figure 13, the fatigue life of weld metal is longer than the base metal under the same plastic strain energy density values. It further illustrates that undermatched welds show better fatigue behaviors than the base metal.

**Figure 14:**
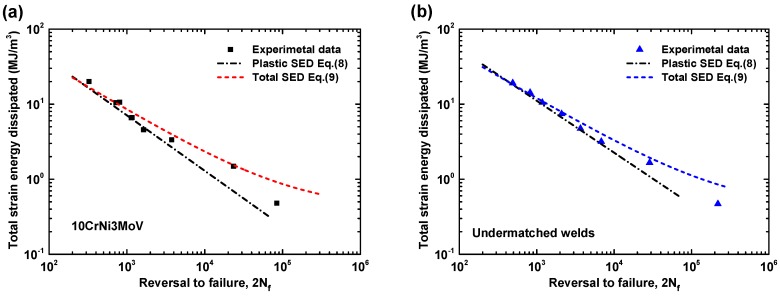
Comparison of total strain energy density Δ*W_t_* from different equations: (**a**) 10CrNi3MoV high strength steel; (**b**) undermatched welds.

In general, the Masing-type Equation (3) for plastic strain energy density is more suitable for the perfect Masing material, such as the base metal 10CrNi3MoV steel. Whereas the predicted Equation (4) for non-Masing material appear to be much accurate than Masing-type Equation (3). A compared summary of experimental data and predicted equations is presented in Table 7. The results are also shown in Figure 14. As can be seen from Figure 14a, the results from predicted equations for 10CrNi3MoV steel are close to the experimental data for high plastic strain energy density. While some deviation appears compared with experimental data in the range of small plastic strain energy density amplitudes. Nevertheless, the results from Masing-assumption model and non-Masing-assumption model are consistency for all the range of plastic strain energy density. Figure 14b shows the comparison of predicted models and experimental data for undermatched welds that reflects a typical non-Masing material behavior. From the results of comparison, the non-Masing material equation is more accurate than the Masing material equation for the weld metal, yet there are close to experimental data.

(8) $$\Delta W_{p}=k_{p}{\left(2N_{f}\right)}^{\alpha_{p}}$$

To reflect the integrity of strain energy under different strain amplitudes, the total strain energy density is computed by the sum of the plastic and the tension elastic strain energy densities of the half-life stress-strain hysteresis loop, from the following Equation (9):

(9) $$\Delta W_{T}=k_{T}{\left(2N_{f}\right)}^{\alpha_{T}}+\Delta W_{0}^{e+}$$

where $k_{T}$ and $\alpha_{T}$ are constants, and $\Delta W_{0}^{e+}$ is the tension strain energy at the material fatigue limit estimated at $2N_{f}=10^{7}$. Finally, the corresponding parameters about plastic strain energy density and total strain energy density are summarized in Table 8.

**Figure 15:**
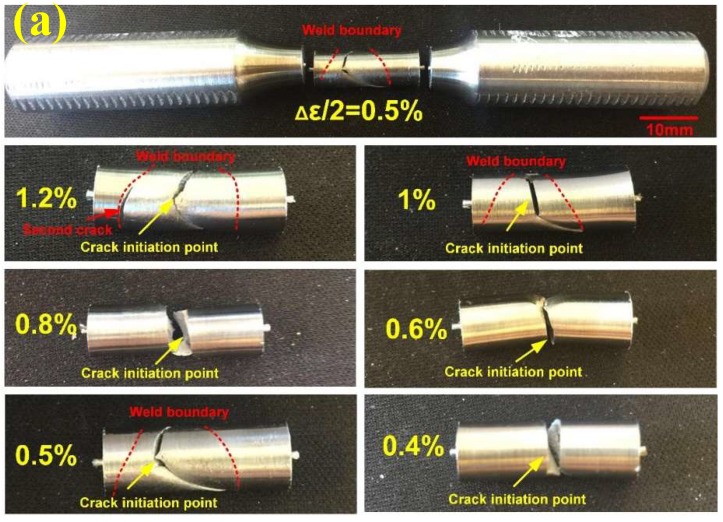

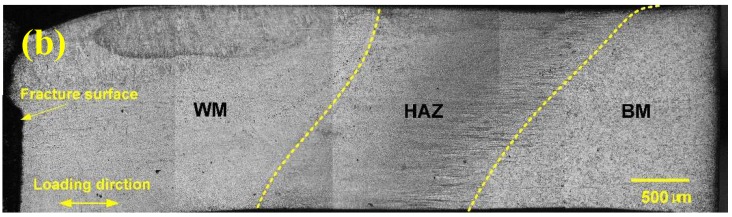
LCF fracture location of the welded joint under different strain amplitudes: (**a**) Macro morphology; (**b**) Optical images for microstructure.

The total strain energy density against the number of reversals to failure for base metal and its undermatched welds are compared in Figure 14. The difference of fatigue behaviors under different total strain energy density amplitudes between the two materials is obvious. Similar with the fatigue assessment of plastic strain energy density, the undermatched weld metal demonstrates better fatigue properties than base metal, even though the cyclic strength of weld metal is lower than base metal. Figure 15 depicts the comparison of total strain energy density $\Delta W_{T}$ based on Equations (8) and (9) for the two materials. The fitting function of Equation (9) has shown good agreement with the experimental observations. Moreover, it is not only suitable for low cycle fatigue but for high cycle fatigue, even can illustrate the mean stress effect. For some complicated components, such as notch components, it has more widely used to assess the fatigue behavior considering stress concentration phenomenon.

### 3.6. The Failure Location of Welded Joints

Due to the discrepancy of the chemical composition and microstructures between the base metal and undermatched weld metal, it is necessary to confirm the fracture location of the undermatched welded joints under low cycle fatigue. It can be seen for all strain amplitudes from Figure 15, the fracture crack is initiated from the undermatched weld metal. During the LCF tests, the strain of the welded joints mainly concentrated on the weld metal (WM) region. Especially at the large strain amplitude (1.2%) from Figure 15a, obvious plastic deformation can be observed in WM region. Although it occurs secondary crack in the HAZ, the final fracture position appears in the weld metal. To further verify fatigue failure locations in low cycle fatigue tested specimens of weld joints, optical metallographic studies had been carried out on longitudinal cross section of the fatigue tested sample (Δ*ε*/2 = 0.6%) in Figure 15b. It can be observed from this figure, the fatigue crack was initiated and propagated in the WM region. It demonstrates again that the weakest region is the weld metal zone.

Since displacement-controlled loading was used for fatigue tests, different peak tensile stresses were observed for weldments and base metal under the same strain amplitude. Due to the lower fatigue strength and fatigue ductility coefficient of the undermatched welds, it is more likely that plastic deformation occurs in these samples. Thus, the final fatigue damage of welds is larger than that of base metal. When the location of fatigue crack initiation is determined, the fatigue life is dependent on the material properties.

### 3.7. The Fatigue Fracture Morphology

**Figure 16:**
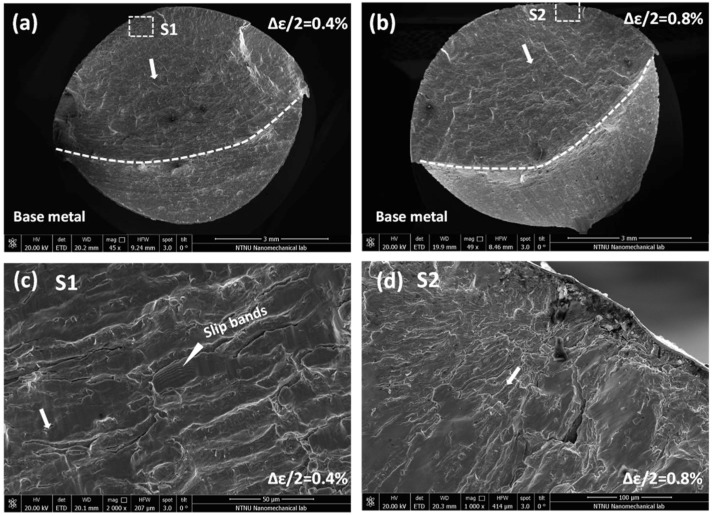
SEM fracture images, under strain amplitudes 0.4% and 0.8% of base metal (10NiCr3MoV). (**a**) over fracture surface under 0.4%; (**b**) over fracture surface under 0.8%; (**c**) crack propagation near crack origin under 0.4%; (**d**) crack origin under 0.4%.

The fractographs of base metal after LCF fatigue tests under 0.4% and 0.8% strain amplitudes are exhibited in Figure 16. Figure 16a (Δ*ε*/2 = 0.4%) shows a fracture surface caused by propagation of cracks nucleated at the specimen periphery. Thus, it leads to some steps in the fracture surface due to the junctions of different propagation planes. In the bottom of this fracture surface, it shows the cleavage steps for final fracture. Figure 16b (Δ*ε*/2 = 0.8%) also gives the fracture surface by propagation of some small cracks, the fracture behaviors of crack nucleation and propagation under Δ*ε*/2 = 0.8% show the similar behavior with Δ*ε*/2 = 0.4%. Normally, the junction under different planes reflects a microscopically inclined fracture. The crack propagation near the nucleation area under Δ*ε*/2 = 0.4% is shown in Figure 16c. Along the direction of crack propagation, ridges appear in propagation regions. The propagation area contains some fatigue striations combined with secondary cracks between large slip bands. These fatigue striations denote essentially transgranular crack propagation. Figure 16d presents the fatigue initiation region under Δ*ε*/2 = 0.8%. The fatigue cracks mainly initiate from the edge of rounding bar. According to the source of cracks, cracks tip propagation pattern is radial. Obvious ridges can be observed from the radial crack.

**Figure 17:**
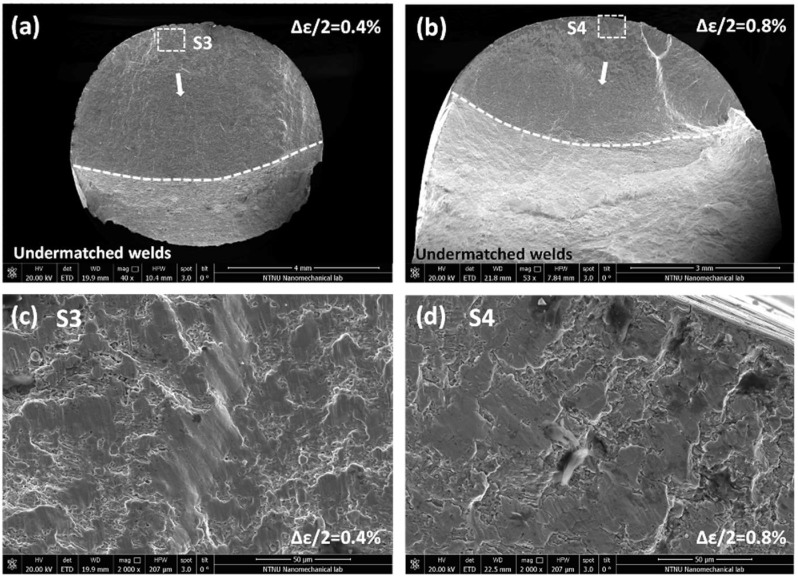
SEM fracture images, under strain amplitudes 0.4% and 0.8% of undermatched welds. (**a**) over fracture surface under 0.4%; (**b**) over fracture surface under 0.8%; (**c**) crack propagation near crack origin under 0.4%; (**d**) crack origin under 0.8%.

Similarly, the fractographs of undermatched welds after LCF fatigue strain amplitudes 0.4% and 0.8% are shown in Figure 17. Fracture surfaces under Δ*ε*/2 = 0.4%, 0.8% initiate from the specimen periphery and propagate along the crack sources. Comparing Figure 17a with Figure 16a, the fracture surface of undermatched welds is smoother than base metal under the same strain loading. No remarkable fatigue striations can be observed by comparing crack propagation details between Figure 17c with Figure 16c. Moreover, it does not appear the second cracks between the flat fatigue bands in the stage of crack propagation. In the stage of crack origination under 0.8% strain amplitude, the same phenomenon shows that the fracture surface of undermatched welds is also more flat than base metal, as shown in Figure 17b. In addition, the microscope pores are found inside of the fractograch, which are formed in the process of melting. Due to the difference of material strength and microstructure, the larger acicular ferrite and granular carbides in base metal facilities the trapping of particles between intercellular spacing than the grain boundaries. Thus, slip bond occurs more steadily across the cells that lead to transgranular cracking. As the fracture path is shorter for transgranular cracking, fatigue resistance can be reduced for base metal. Therefore, the undermatched welds demonstrate better fatigue ductility longer fatigue life than base metal.

## 4. Conclusions

The low cycle fatigue behaviors of 10CrNi3MoV high strength steel and its corresponding undermatched welds were evaluated by means of experimental tests. A series of strain-controlled fully-reversed fatigue tests were conducted under strain amplitudes ranging from 2–1.2% by smooth specimens. The cyclic deformation response, fatigue strength, fatigue ductility properties and cyclic strain energy density were analyzed. The following conclusion can be drawn as follows:

(1) The cyclic strength mismatch ratio showed some discrepancy with the mismatch ratio under monotonic loading for these materials.
(2) A gradual cyclic softening behavior under different strain amplitudes was observed for the two materials. Moreover, the soften behavior mainly appeared in the beginning cyclic stage, which took nearly 5–15% of fatigue life ratio.
(3) The fatigue results show low strength weld metal exhibit a higher fatigue resistance than 10CrNi3MoV steel for all the range of total strain amplitudes, it illustrates that the enhancement of material strength cannot guarantee the proper improvement of fatigue properties.
(4) According to the hysteresis loops under different strain amplitudes, 10CrNi3MoV high strength steel demonstrated almost ideal Masing-type behavior, whereas the undermatched weld metal exhibited non-Masing-type behavior.
(5) The relationship between plastic strain energy density at half-life cycle against the number of reversals to failure is fitted satisfactorily by the power-low equation. The total strain energy density is an adequate parameter for both high- and low-cycle fatigue regimes.
(6) The fatigue assessment for these two materials based on the plastic and total strain energy density all shows that the undermatched weld metal has better fatigue resistance than base metal.

## Figures and Tables

**Table 1:** Comparison of the chemical composition between 10CrNi3MoV steel and under-matched welds.

Steel	C (%)	Si (%)	Mn (%)	Cr (%)	Mo (%)	Ni (%)	Cu (%)	V (%)	S (%)	P (%)
10CrNi3MoV	0.09	0.29	0.48	0.94	0.4	2.88	-	0.06	0.005	0.011
U-Welds	0.027	0.243	1.3	0.051	-	1.09	0.05	-	0.0073	0.011

**Table 2:** Welding conditions of multi-pass plate for the fatigue specimens.

Current	Voltage	Welding Speed	Electrode Diameter	Shielding Gas 80%Ar-20%CO_2_	Heat Input	Interpass Temperature
(A)	(V)	(mm/s)	(mm)	(L/min)	(KJ/mm)	(°C)
140–190	24–28	4.5–5.3	1.2	20	0.7–0.85	<80

**Table 3:** Mechanical properties of test materials.

Steel	Yield Strength (MPa)	Tensile Strength (MPa)	Young’s Modulus (GPa)	Poisson’s Ratio	Kv (J) −20 °C
10CrNi3MoV	693	741	205	0.3	280
U-Welds	498	559	195	0.3	260

**Table 4:** Summary of LCF fatigue tests results.

Specimens Reference	Total Strain Amplitude, Δ*ε*/2 (%)	Elastic Strain Amplitude, Δ*ε_e_*/2 (%)	Plastic Strain Amplitude, Δ*ε_p_*/2 (%)	Stress Amplitude, Δ*σ*/2 (MPa)	Plastic Strain Energy Density Δ*W_p_* (MJ/m^3^)	Total Strain Energy Density Δ*W_T_* (MJ/m^3^)	Number of Cycle to Failure, *N_f_*
BM1	1.2	0.469	0.731	595	18.546	19.940	163
BM2	0.8	0.296	0.504	566	9.667	10.487	361
BM3	0.8	0.290	0.510	565	9.799	10.618	405
BM4	0.6	0.291	0.309	567	5.787	6.613	585
BM5	0.6	0.286	0.314	561	5.759	6.561	571
BM6	0.5	0.280	0.220	537	3.812	4.564	820
BM7	0.4	0.331	0.069	510	2.500	3.345	1878
BM8	0.3	0.280	0.021	495	0.795	1.487	11,737
BM9	0.2	0.200	-	480	0	0.480	42,146
WM1	1.2	0.276	0.924	542	18.377	19.125	245
WM2	1	0.254	0.746	511	13.593	14.242	410
WM3	0.8	0.261	0.539	535	9.801	10.499	590
WM4	0.6	0.247	0.353	489	6.807	7.410	1048
WM5	0.5	0.298	0.202	435	4.084	4.733	1838
WM6	0.4	0.215	0.185	424	2.719	3.175	3412
WM7	0.3	0.198	0.102	386	1.181	1.662	14,389
WM8	0.21	0.2	-	370	0	0.470	109,640

**Table 5:** Mechanical properties of test materials.

Mechanical Properties	10CrNi3MoV	Undermatched Welds
Young’s modulus (GPa)	205	195
Cyclic hardening coefficient, *K*′ (MPa)	857.16	1251.8
Cyclic hardening exponent, *n*′	0.079	0.172
Master curve hardening coefficient, K* (MPa)	1113	685.99
Master curve hardening exponent, n*	0.112	0.079

**Table 6:** Fatigue strength and fatigue ductility parameters of 10CrNi3MoV high strength steel and undermatched welds.

Mechanical Properties	10CrNi3MoV	Undermatched Welds
Fatigue strength coefficient, $\sigma_{f}^{\prime }$	1386.4	896.9
Fatigue strength exponent, *b*	−0.108	−0.067
Fatigue ductility coefficient, $\varepsilon_{f}^{\prime }$	0.779	0.5351
Fatigue ductility exponent, *c*	−0.798	−0.65

**Table 7:** Experimental and theoretical Δ*W_p_* values of 10CrNi3MoV high strength steel and undermatched welds.

Specimens Reference	Total Strain Amplitude, Δ*ε*/2 (%)	Plastic Strain Energy Density Δ*W_p_* (MJ/m^3^) from Experiments	Plastic Strain Energy Density Δ*W_p_* (MJ/m^3^) from Equation (3)	Plastic Strain Energy Density Δ*W_p_* (MJ/m^3^) from Equation (4)
BM1	1.2	18.546	14.850	13.599
BM2	0.8	9.667	9.740	8.909
BM3	0.8	9.799	9.838	8.999
BM4	0.6	5.787	5.982	5.472
BM5	0.6	5.759	6.014	5.500
BM6	0.5	3.812	4.034	3.685
BM7	0.4	2.500	1.201	1.096
BM8	0.3	0.795	0.355	0.324
BM9	0.2	0	0	0
WM1	1.2	18.377	14.153	17.943
WM2	1	13.593	10.773	13.697
WM3	0.8	9.801	8.149	10.338
WM4	0.6	6.807	4.878	6.216
WM5	0.5	4.084	2.483	3.185
WM6	0.4	2.719	2.217	2.847
WM7	0.3	1.181	1.113	1.437
WM8	0.21	0	0	0

**Table 8:** Energy-based properties of 10CrNi3MoV high strength steel and undermatched welds.

Mechanical Properties	10CrNi3MoV	Undermatched Welds
$k_{p}$ (MJ/m^3^)	1162.1	1318.8
$\alpha_{p}$	−0.738	−0.69
$k_{T}$ (MJ/m^3^)	599.6	751.8
$\alpha_{T}$	−0.622	−0.603
$\Delta W_{0}^{e+}$	0.213	0.382
